# Outcomes of proximal humeral chondroblastoma treated with intralesional curettage, electrocauterization of the cavity and bone grafting: A retrospective study

**DOI:** 10.1371/journal.pone.0354589

**Published:** 2026-07-27

**Authors:** Jingjing Zuo, Lijun Liu, Xueyang Tang, Lei Yang

**Affiliations:** 1 Rehabilitation Medicine Centre, West China Hospital, Sichuan University, Chengdu, Sichuan, People’s Republic of China; 2 Department of Pediatric Surgery, West China Hospital, Sichuan University, Chengdu, Sichuan, People’s Republic of China; Menoufia University, EGYPT

## Abstract

**Objectives:**

This study aimed to evaluate the 7-year outcomes of proximal humeral chondroblastoma treated with intralesional curettage, electrocauterization of the cavity and bone grafting in pediatric patients.

**Methods:**

A total of 17 patients diagnosed with proximal humeral chondroblastoma who underwent surgical treatment consisting of intralesional curettage, cavity electrocauterization, and bone grafting at our center between January 1, 2008, and January 1, 2018, were retrospectively enrolled. All patients received postoperative splint immobilization for four weeks and were followed up for a minimum of seven years. Tumor recurrence was evaluated based on clinical manifestations and radiographic X-ray examination. Clinical outcomes included functional performance assessed using the Musculoskeletal Tumor Society (MSTS) score and the Quick Disabilities of the Arm, Shoulder and Hand (Quick DASH) scale, as well as postoperative complications.

**Results:**

In total, 17 patients with proximal humeral chondroblastoma were enrolled in our study, including 10 males and 7 females, with a mean age of 10.9 ± 1.8 years old (range from 8 to 14 years). Among these 17 cases, there were 6 cases with lesions extending through the epiphyseal plate, and 11 cases with lesions located inside the epiphysis. During follow-up, 1 patient suffered reoperation due to tumor recurrence. Shortening of the affected upper limb occurred in 9 patients (52.9%), which was significantly associated with a young age(P = 0.012). The mean shortening was 1.2 ± 0.9 cm compared with the contralateral limb. The overall mean postoperative MSTS was 27.5 ± 1.4 points, with no significant difference between observed the limb shortening group (27.3 ± 1.4) and the non-shortening group (27.8 ± 1.4,P = 0.550). The overall Quick DASH score averaged 4.8 ± 3.9 (0–15.9), and no statistically significant difference was detected between the shortening group (5.3 ± 4.5) and the non-shortening group (4.3 ± 2.8; P = 0.452).

**Conclusions:**

Intralesional curettage, cavity electrocauterization and bone grafting is an optional treatment for children with proximal humeral chondroblastoma but still has a risk of recurrence. Shortening of the affected upper limb is a common complication, which is possibly associated with young age but has no impact on the final functional outcomes.

**Level of Evidence:**

Level IV, retrospective case series.

## Introduction

Chondroblastoma is a rare bone tumor that mainly occurs in young people. Although it is considered a benign tumor, it still causes damage to the epiphyses of long bones and has a low risk of lung metastasis [1]. The proximal humerus has been reported as the most common location affected by chondroblastoma [2]. Clinical manifestations of the disease are not typical, including pain, swelling, limited motion of the joint and joint effusion, which could also present in other bone tumors [3]. The primary treatment of chondroblastoma is curettage and bone grafting according to the defect [4]. Some authors supported local chemical or physical adjuvant therapy after surgical curettage to decrease tumor recurrence [5]. Despite the efforts on adjuvant therapy, there is still a risk of local recurrence as high as 30% [6,7]. The functional outcomes are generally good [2], especially for those affecting proximal humerus due to the compensatory effect of the upper limbs.

The aim of this study was to retrospectively evaluate the clinical outcomes of proximal humeral chondroblastoma treated with intralesional curettage, electrocauterization of the cavity and bone grafting in our center. The details of the patients’ general data and clinical results are presented here.

## Patients and methods

We searched the diagnosis card index files at our department in West China Hospital. Data were collected from patients diagnosed with proximal humeral chondroblastoma between 01/01/2008 and 01/01/2018. A total of 18 patients were identified and pathologically confirmed. One patient was excluded due to loss to follow-up, and 17 patients with a minimum follow-up of 7 years were finally enrolled in this study. This study was reviewed and approved by the Ethics Committee for Medical Research at West China Hospital, Sichuan University in accordance with the Declaration of Helsinki (Committee’s reference number: 2024(2231), Chengdu, Sichuan, 2024). Informed consent was obtained from all patients for being included in the study, and written informed consent regarding publishing their data and photographs was obtained from parents of all pediatric participants and approved by the Institutional Review Board. The individual in this manuscript has given written informed consent to publish these case details.

**Fig 1:**
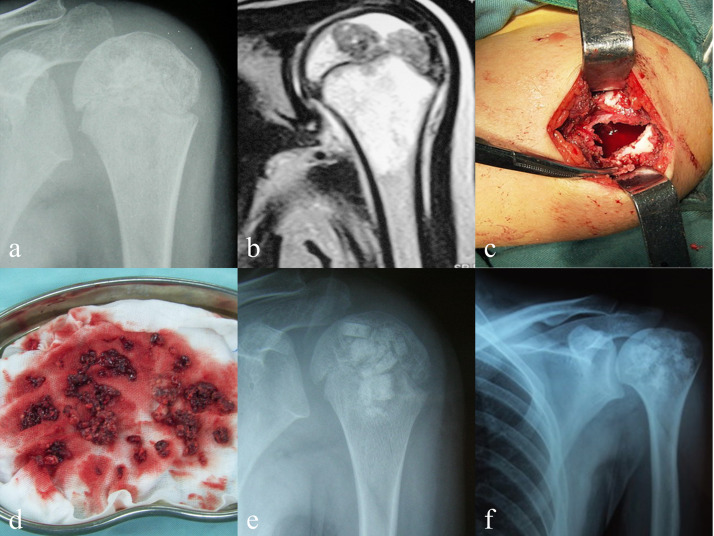
An 11-year-old boy with chondroblastoma involving the proximal humerus of the left side treated with intralesional curettage, electrocauterization of the cavity and bone grafting. **a, b** Radiographs showing eccentric round-like focus located in the epiphysis with a lytic lesion on X-ray film and Magnetic Resonance Imaging scan. **c** Intraoperative photo after curettage (the proximal humerus). **d** Gross appearance of pathological tissues. **e** Postoperative radiograph showing the lesions after curettage and grafting. **f** Radiograph at the 8-year follow-up.

Surgeries were performed under general anesthesia by two senior pediatric orthopedic surgeons well trained in bone tumor treatment. All 17 patients received open surgery for intralesional curettage, electrocauterization of the lesion cavity and bone grafting. First, the visualized gross tumor was removed with a curette, and then a high-speed burr was used to grind the tumor cavity. The tumor cavity curettage was confirmed by fluoroscopy and endoscope, and then followed by the cavity electrocauterization with an electrotome in the spray coagulation mode at 80 W and confirmed by an endoscope. Finally, bone grafting was performed based on the size of the defect and location. 15 patients received autogenous iliac bone grafting, 2 patients received autogenous iliac bone and artificial bone due to the large defect after curettage (Fig 1).

Postoperatively, the extremity was placed in a well-padded splint for at least 4 weeks with no weight bearing. All patients were followed up for at least 7 years with a mean follow-up of 8.9 ± 1.6 years, and complete physeal closure was confirmed in every patient during the follow-up. Patients were followed up every two weeks in the first 2 months after surgery, then every 2 months during the first year, every six months in the second year and every 2 years after the second year.

The clinical outcomes included functional outcomes evaluating by using the Musculoskeletal Tumor Society Score (MSTS) and Quick DASH scale [6], tumor recurrence and complications such as upper limb shortening. Local recurrence was examined by clinical manifestation and X-ray, and lung metastasis was examined by chest X-ray. One pediatric rehabilitation therapist and one pediatric orthopedic surgeon independently conducted the MSTS and Quick DASH evaluation in a blinded manner at the 7-year follow-up after treatment. Upper limb length was measured using superficial anatomical landmarks from the acromion to the lateral humeral epicondyle. Two independent pediatric orthopedic surgeons performed all clinical measurements in a blinded and independent fashion. The statistical analysis confirmed high inter-rater agreement. Radiographic measurement of the contralateral limb was not adopted, as it is clinically unreasonable to expose patients to unnecessary additional radiation. Upper limb shortening was defined as a length discrepancy of ≥1 cm relative to the contralateral side.

SPSS 20 was used for data analysis. Continuous data were reported using the mean ± standard deviation (SD) with range and categorical data are reported as numbers and percentages. Comparisons of continuous data between the limb-shortening and non-shortening groups were analyzed by independent-samples T test. Comparisons between preoperative and postoperative continuous data were analyzed by paired-samples T test. Fisher’s exact test was used for all categorical comparisons. A value of P < 0.05 was considered statistically significant.

## Results

In total, 17 patients (10 male and 7 female) were enrolled in our study, with a mean age of 10.9 ± 1.8 years old (range from 8 to 14 years). The detailed data are presented in Table 1. The major clinical manifestations included 17 cases of pain, 3 cases of local swelling, 6 cases of limited range of motion of the adjacent joints. The mean duration of symptoms was 6.7 ± 2.8 months (range from 2–12 months). Only 12 patients were considered diagnoses of chondroblastoma preoperatively according to the typical characteristics of chondroblastoma (based on symptoms, signs, and imaging examinations), and the other 5 patients were undiagnosed preoperatively. All patients underwent biopsy prior to definitive surgery.

**Table 1:** The detailed data of the patients and univariate analysis of risk factors for upper limb shortening.

	Limb shortening (n = 9)	Non-shortening (n = 8)	P
Gender			1.000
Female	4(23.5%)	3(17.6%)	
Male	5(29.4%)	5(29.4%)	
Mean age, years	9.9 ± 1.5	12.0 ± 1.5	**0.012**
Side			0.335
Left	4(23.5%)	6(35.3%)	
Right	5(29.4%)	2(11.8%)	
Epiphyseal plate involved			0.335
Yes	2(11.8%)	4(23.5%)	
No	7(41.2%)	4(23.5%)	
Artificial bone grafting			0.735
Yes	1(5.9%)	1(5.9%)	
No	8(47.0%)	7(41.2%)	
MSTS	27.3 ± 1.4	27.8 ± 1.4	0.550
Quick DASH	5.3 ± 4.5	4.3 ± 2.8	0.586

Bold values are statistically significant p < 0.05. MSTS means Musculoskeletal Tumor Society Score. Quick DASH means Quick Disabilities of the Arm, Shoulder, and Hand questionnaire.

Postoperatively, one patient developed a surgical wound infection with a delay of incision healing after treatment with antibiotics and subcutaneous drainage. One patient (1/17, 5.9%; 95% CI: 0.1%–28.7%) developed local recurrence 9 months after the initial treatment of curettage, electrocauterization and autogenous iliac bone graft. This patient received a reoperation of extended lesion curettage. Lung metastasis was not observed in this group of patients.

The mean length of the affected upper limb was 28.7 ± 1.6 cm, with a mean shortening of 1.2 ± 0.9 cm compared with the contralateral limb. Shortening of the affected upper limb occurred in 9 patients (52.9%), which was associated with young age(P = 0.012). Since no obvious functional impairment was noted, no additional treatment was administered.The mean age of the upper limb shortening group was 9.9 ± 1.5 years old and that in the non-shortening group was 12.0 ± 1.5 years old. There were 6 cases with lesions extending through the epiphyseal plate and the other 11 cases with lesions located inside the epiphysis. The mean proportion of the epiphyseal plate affected by the lesion (calculated as lesion radius divided by the minor axis of the epiphyseal plate) was 0.347 ± 0.169. The relative extent of the tumor extending distal to the growth plate (length of the distal tumor component divided by the total lesion length on coronal MRI) was 0.185 ± 0.123.

The mean postoperative MSTS was 27.5 ± 1.4 points and 17 patients gained good functional outcomes. Despite the complication of limb shortening, there was no significant difference in MSTS between the limb shortening group (27.3 ± 1.4) and the non-shortening group (27.8 ± 1.4, P = 0.550). The overall Quick DASH score averaged 4.8 ± 3.9 (0–15.9). No statistically significant difference was found between the limb shortening group (5.3 ± 4.5) and the non-shortening group (4.3 ± 2.8; P = 0.452).

## Discussion

Chondroblastoma usually arises during the teenage years of young people [8] and mainly affects the epiphyses of long bones, such as the humerus, femur and tibia. Less common locations have also been reported in the literature, including the patella, calcaneus, acetabulum and spine [9–11]. It seems that chondroblastoma occurs more frequently in males than females, with a ratio of 2.3 to 1 in the literature [2,12]. In our study of chondroblastoma, the mean age of onset was 10.9 ± 1.8 years old with a male to female ratio of 1.4:1, which all coincided with most previous studies. This group of patients all have the symptom of pain with an average time of observation nearly 6 months, mainly due to our strictly controlled surgical indications and concerns on physeal injury.

The clinical manifestation of chondroblastoma is not typical and the preoperative diagnosis is difficult [4]. Therefore, a characteristic imaging finding may be very helpful for diagnosis, including an eccentric round-like focus located in the epiphysis with a lytic lesion, calcification or a peripheral sclerotic margin and usually a significantly higher-intensity signal on T2-weighted MRI [13,14]. El-Ali AM et al [15] reported that chondroblastoma was one of the most common diseases presenting as solitary long-bone epiphyseal lesions and was characterized by a peripheral location, discrete T1-weighted hypointense rim. Blancas C et al [16] also found these common imaging appearances as well as periosteal reaction and perilesional edema in chondroblastoma. Plain radiographs provide rapid and intuitive evaluation of bony lesions and osseous morphological changes, while it remains challenging to reach a precise clinical diagnosis based merely on radiographs, patient history and physical findings. MRI offers superior soft-tissue resolution, accurately delineates epiphyseal plate involvement, tumor boundary, and intramedullary extension, which is critical for surgical strategy formulation and risk assessment of growth disturbance. Postoperative limb deformities can be comprehensively evaluated using plain radiographs, which clearly demonstrate the overall morphological abnormalities. MRI serves as the primary imaging modality for assessing detailed epiphyseal plate status and identifying physeal injury or bony bridge formation. In this group of patients, the major clinical manifestations was pain in the shoulder area which was atypical and helped little for diagnoses. The preoperative diagnoses of these 12 patients were mainly based on the imaging characteristics. Therefore, patients with these typical imaging features, lesion locations and the appropriate age should be considered the possibility of chondroblastoma [17].

Surgical removal and bone grafting are still the standard treatments for chondroblastoma [1,2]. Some authors advise adjuvant physical or chemical inactivation methods after a complete curettage to lower the recurrence, including high-speed burr, electrocauterization, phenol, cryotherapy, ethanol and others [5,18,19]. Ebeid WA et al [4] reported a significantly lower recurrence rate in patients treated by thorough curettage and high-speed burr physical inactivation. Zekry KM et al [20] reported that extended curettage with phenol and ethanol as local adjuvants was effective in lowering the incidence of local recurrence. The physical adjuvants of high-speed burr plus electrocautery offers the advantages of precise intraoperative manipulation, minimal damage to adjacent deep tissues, and extensive tumor removal. Furthermore, the entire procedure can be precisely monitored under endoscopic visualization. Nevertheless, all these adjuvant therapies may cause extensive tissue damage and therefore require cautious application, particularly for physis-involved tumors [5]. Notably, no previous studies have comprehensively compared the efficacy and safety of different physical or chemical adjuvant treatments for chondroblastoma.

Tumors involving the physis in immature skeleton patients might cause growth disturbance, which is also a concern during treatment [6]. Some authors believe that complete lesion curettage to lower local recurrence is more important than the protection of the epiphyseal plate [20]. Mashhour and Abdel Rahman [21] preferred aggressive surgical curettage at the initial treatment to prevent recurrence, and they thought that reoperation after recurrence might cause more damage to the growth plate. Liu Q et al [22] also reported the aggressive curettage with phenolization adjuvant and allogeneic bone grafting as an effective treatment for chondroblastoma in young patients. Xiong Y et al [23] found that the lesion crossing the epiphyseal line led to more complications, while a thorough curettage was still necessary and the final functional outcomes were still good. Muratori F et al. [24] also reported the complication of limb shortening among patients with tumor involvement of the growth plate. The physeal damage may result from tumor invasion and aggressive curettage is the mainstay of treatment for chondroblastoma. In this descriptive observation, lesions involving the epiphyseal plate did not correspond to a higher risk of limb shortening. This trend may be partially related to the older age of patients in this small and unbalanced subgroup.

Despite the high risk of complications, the functional outcomes are generally good [25]. In a retrospective study by Farfalli GL et al [26], the local complications was observed in 43% of all the cases. While the mean MSTS score in their study was 28 of 30 points, and was considered to be excellent. Xiong Y et al [23] reported the common complication of limb length discrepancy in their retrospective study, but showed no impact on the functional outcomes. We also found no relation between the upper limb shortening and final function (P = 0.550), which may be the reason of the strong compensatory effect of the upper limb [27].

The prognosis of chondroblastoma is generally good [4], while there are still risks of local recurrence and extremely rare lung metastasis [28,29]. Age, location, persistent physis and inadequate curettage were reported as possible risk factors in some literature but are still lack reliable evidence [7,13,25]. Ebeid WA et al [4] reviewed 91 patients with chondroblastoma and found no statistical significance between local recurrence and factors of age, sex, pathologic fracture and the types of filler used. Outani H et al [30] reported a 20% (8 cases)recurrence rate in their retrospective study of 40 patients treated with synthetic bone substitute and intralesional curettage, while no reliable factors for recurrence could be concluded. Nevertheless, most authors still consider inadequate curettage as a possible risk factor associated with recurrence and preferred a thorough curettage in treatment [2,4,6,31].

Some limitations of our study included that it was a retrospective case series study in a single center, and it had a small sample size with no control. The relatively small sample size and absence of a control group are inevitable given the extreme rarity of proximal humeral chondroblastoma. This limitation also applies to the subgroup related to the epiphyseal plate, sex, or age. Thus, these findings are descriptive rather than confirmatory. Individual patient-level data cannot be provided due to restrictions on data access for the protection of pediatric participants’ privacy. There is still a need for further multicenter studies with large samples, especially in terms of adjuvant treatment options and risk factors for recurrence.

## Conclusions

Intralesional curettage, cavity electrocauterization and bone grafting is an optional treatment for children with proximal humeral chondroblastoma but still has a risk of recurrence. Postoperative ipsilateral upper limb shortening is a common complication that is likely correlated with young patient age; however, such limb length discrepancy does not adversely affect long-term upper limb functional outcomes.

## Abbreviations

MSTS: Musculoskeletal Tumor Society Score
CT: computed tomography
MRI: magnetic resonance imaging
